# External Quality Assessment as a strategy for Chagas disease surveillance: a study in four municipalities of Pernambuco

**DOI:** 10.1590/0037-8682-0361-2025

**Published:** 2026-02-16

**Authors:** Efraim Naftali Lopes Soares, Dayse da Silva Rocha, Allan Pitta Seabra, João Paulo Sales Oliveira-Correia, Fernando Vinicius Gomes de Moraes, Rafaella Albuquerque e Silva, Cleber Galvão

**Affiliations:** 1 Secretaria de Saúde de Caruaru, Caruaru, PE, Brasil.; 2 Fundação Oswaldo Cruz, Instituto Oswaldo Cruz, Laboratório Nacional e Internacional de Referência em Taxonomia de Triatomíneos, Rio de Janeiro, RJ, Brasil.; 3 Secretaria de Vigilância em Saúde, Brasília, DF, Brasil.

**Keywords:** Entomological surveillance, Reference, Taxonomy, Triatominae

## Abstract

**Background:**

Triatomines are hematophagous insects that act as vectors of *Trypanosoma cruzi*, the causative agent of Chagas disease. External Quality Assessment (EQA) is a tool used for monitoring and improving taxonomic identification activities performed by the health laboratories in Brazil.

**Methods:**

EQA were conducted in four municipalities in the state of Pernambuco. Boxes containing a panel of 12 adult Hemiptera specimens (male and female) were sent for species identification.

**Results:**

The results revealed 68% accuracy, with difficulty for morphologically similar species, such as those of the genus *Rhodnius*.

**Conclusions:**

EQA is essential for monitoring laboratory performance, highlighting the need for ongoing training.

Triatomines are hematophagous insects that act as vectors for *Trypanosoma cruzi* (Chagas, 1909), the causative agent of Chagas disease^1^. This disease is endemic to the Americas, present in 21 countries and affects more than seven million people, with approximately 100 million individuals at risk of infection^2^.

Currently, 159 species of triatomines are known, of which 64 are widely distributed in Brazil, with 14 recorded in the state of Pernambuco: *Panstrongylus geniculatus* (Latreille, 1811), *Panstrongylus megistus* (Burmeister, 1835), *Panstrongylus lutzi* (Neiva & Pinto, 1923), *Panstrongylus tibiamaculatus* (Pinto, 1926), *Rhodnius nasutus* Stål, 1859, *Rhodnius neglectus* Lent, 1954, *Triatoma brasiliensis* Neiva, 1911, *Triatoma infestans* (Klug, 1834), *Triatoma melanocephala* Neiva & Pinto, 1923, *Triatoma pseudomaculata* Corrêa & Espínola, 1964, *Triatoma petrocchiae* Pinto & Barreto, 1925, *Triatoma rubrofasciata* (De Geer, 1773), *Triatoma sordida* (Stål, 1859) and *Psammolestes tertius* Lent & Jurberg, 1965^3^. Although all known species are considered potential vectors of *T. cruzi*, only a few have epidemiological importance, such as *T*. *brasiliensis* and *T. pseudomaculata* in Pernambuco^4^. However, owing to the advancement of deforestation, anthropogenic actions, and climate change, species previously considered secondary, such as *P. lutzi*, may become more relevant in the transmission dynamics of Chagas disease^1^ ^,^ ^5^.

External Quality Assessment (EQA) is a fundamental tool for monitoring and improving taxonomic identification in public health laboratories in Brazil. Accurate identification of triatomines is essential in the context of Chagas disease control to guide entomological surveillance strategies and vector control actions^6^. 

The municipalities of Caruaru (Agreste Pernambucano), Salgueiro (Sertão Central), Arcoverde (Sertão do Moxotó) and Afogados da Ingazeiras (Sertão do Pajeú) are located in the inland region of the state of Pernambuco^7^. These areas have a long history of triatomine occurrence, placing their populations at continued risk of vector-borne transmission of Chagas disease^8^. Triatomines of great epidemiological importance have been recorded in these municipalities, mainly *T. brasiliensis*, *T. pseudomaculata* and *P. lutzi.* These species find favorable conditions in peridomestic ecotopes, such as chicken coops, lumberyards, and rural outbuildings, in addition to occupying natural shelters in the caatinga areas^5^. 

The vulnerability of rural populations to contact with triatomines makes these municipalities important for Chagas disease surveillance. Correct identification of triatomines in these locations is essential for guiding entomological surveillance efforts, preventing the establishment of domestic colonies, and reducing the risk of vector-borne transmission of Chagas disease^8^. 

In 2025, the External Quality Assessment of Triatomines (EQA-T), coordinated by the Laboratório Nacional e Internacional de Referência em Taxonomia de Triatomíneos (LNIRTT/Fiocruz), aims to evaluate the accuracy of taxonomic identification performed by laboratory teams in the municipalities of Caruaru, Salgueiro, Arcoverde, and Afogados da Ingazeira in Pernambuco. The assessment highlighted the weaknesses of the identification process, discussed the importance of continuous training of professionals involved in triatomine identification, and indicated pertinent corrective actions to strengthen entomological surveillance.

To conduct the EQA, six laboratories from four municipalities were invited by the LNIRTT (through an invitation letter) to participate voluntarily in the study (Figure 1). After agreeing to voluntary participation, the laboratories were provided with a Free and Informed Consent Form (FIC). 

**FIGURE 1:**
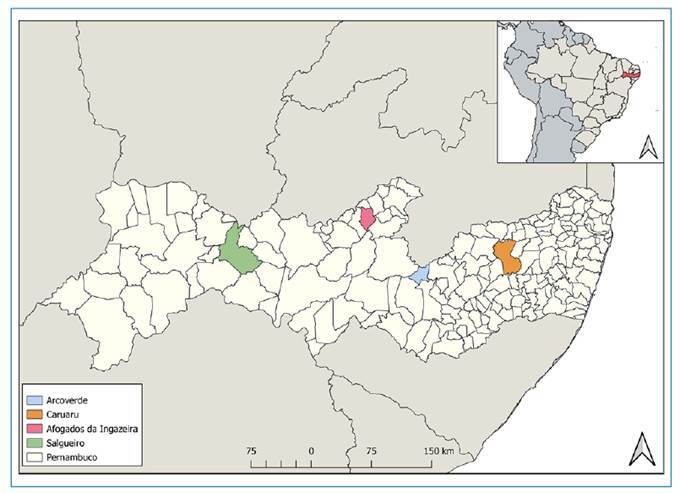
Location of participating laboratories in the State of Pernambuco, 2025.

Each laboratory received a panel consisting of 12 adult Hemiptera specimens (male and female), accompanied by the book “*Vetores da Doença de Chagas no Brasil*”^9^ ^,^ which contains dichotomous keys for classification at the species level, a form for submitting results, and instructions on how to complete the evaluation (Figure 2). The participants were guaranteed impartiality, independence, and confidentiality.

**FIGURE 2:**
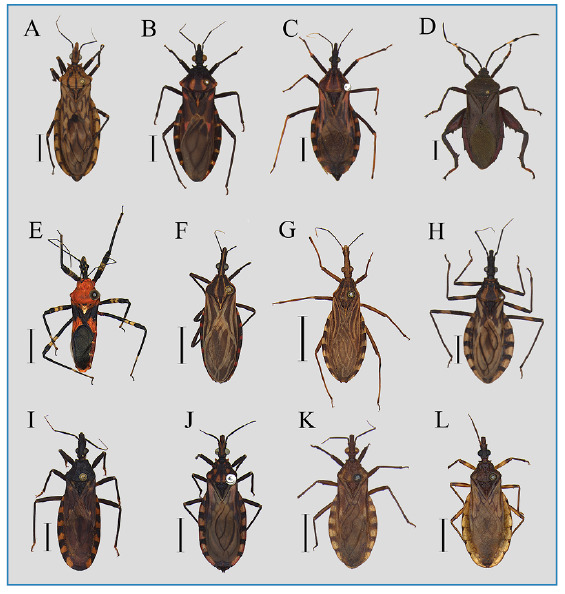
In dorsal view: **(A)** *Panstrongylus lignarius*, **(B)** *Panstrongylus megistus*, **(C)** *Panstrongylus tibiamaculatus* (*Triatoma tibiamaculata*), **(D)** Phytophagous, **(E)** Predator, **(F)** *Rhodnius brethesi*, **(G)** *Rhodnius neglectus*, **(H)** *Triatoma brasiliensis*, **(I)** *Triatoma infestans*, **(J)** *Triatoma maculata*, **(K)** *Triatoma pseudomaculata*, **(L)** *Triatoma sordida*. Scale in 5 mm.

In addition to species-level identification, the form addressed the condition of the material received, integrity of the specimens upon receipt, condition of the laboratory equipment used, sex of the specimens, and any difficulties encountered during identification. The deadline for the submission of the results was seven days after receiving the panel.

Each panel contained ten species of triatomines (both sylvatic and domestic), one phytophagous hemipteran, and one predator. Triatomine species include *T. infestans*, *T. brasiliensis*, *T. pseudomaculata*, *Triatoma maculata* (Erichson, 1848), *T. sordida*, *R. neglectus*, *Rhodnius brethesi* Matta, 1919, *P. megistus*, *Panstrongylus lignarius* (Walker, 1873), and *Panstrongylus tibiamaculatus* (Pinto, 1926). To facilitate the use of the available dichotomous keys, *P. tibiamaculatus* was maintained in the genus *Triatoma* (i.e., *T. tibiamaculata*), as there were no updated keys reflecting its current taxonomic *status*.

Six of the selected triatomine species are known to occur in Pernambuco, including the two with greatest epidemiological importance (*T. brasiliensis* and *T. pseudomaculata*), two species are recorded in the North region of Brazil (*T. maculata* and *R. brethesi*), one species (*P. lignarius*) is found in only one northeastern state (Maranhão) with a greater distribution in the North region (Amazonas, Pará, and Tocantins), and *T. infestans* is currently restricted to residual foci in two Brazilian states, Bahia and Rio Grande do Sul^10^.

All specimens were examined individually using dichotomous keys, with special attention paid to the morphological characteristics essential for correct species identification. The insects were randomly mounted on styrofoam plates using entomological pins, labeled with letters (A to L), and then packed in boxes for shipment to the participating laboratories. A template was established randomly for each laboratory. The shipment was made using MedicalBox for biological substances and transported by a specialized company.

All the laboratories used a stereoscopic microscope and met the deadline for submitting responses, which was also considered a factor in the performance evaluation.

The results submitted by the participating laboratories were compared with the templates held by the laboratory responsible for coordinating the study and were scored as correct or incorrect. Any absence of a response was considered incorrect when calculating the proportion of correct responses.

A considerable variation in performance was observed among the participating laboratories, with a correct response rate of 68% (49/72). The species with the most correct identifications were *T. tibiamaculata*, *P. lignarius* and *T. sordida*, which were correctly identified by five of the six laboratories, even when the specimens presented damage to their morphological structure. The laboratories reported that 33% of the insects had some damage to their morphological structure (24/72). The compromised structures were the antennae and legs, with only one insect presenting damage to its wings **(** Table 1 **)**.

**TABLE 1:** Laboratory responses in the identification of triatomines.

Taxa	Laboratories											
	1		2		3		4		5		6	
	Matches	Damages	Matches	Damages	Matches	Damages	Matches	Damages	Matches	Damages	Matches	Damages
Phytophagous	+	N	+	N	-	N	-	N	+	N	+	N
Predator	+	Y^3^	+	Y^4^	-	N	+	N	+	N	-	N
*Panstrongylus lignarius*	+	Y^1^	+	Y^2^	+	Y^1^	-	N	+	N	+	N
*Panstrongylus megistus*	+	Y^3^	-	Y^2^	+	Y^1^	-	N	+	N	+	N
*Rhodnius brethesi*	+	N	+	N	-	N	+	N	+	N	-	N
*Rhodnius neglectus*	-	Y^1^	+	Y^3^	-	N	-	N	+	N	+	N
*Triatoma brasiliensis*	-	N	-	N	+	N	+	N	+	N	+	N
*Triatoma infestans*	+	Y^1^	-	Y^1^	-	Y^4^	+	N	-	N	+	N
*Triatoma maculata*	+	Y^1^	+	Y^3^	-	N	+	N	+	Y^3^	+	N
*Triatoma pseudomaculata*	+	N	-	Y^3^	+	N	+	N	+	N	-	N
*Triatoma sordida*	+	Y^1^	+	N	+	Y^1^	+	N	-	N	+	N
*Triatoma tibiamaculata*	+	Y^1^	+	Y^3^	+	Y^1^	-	N	+	N	+	N

**Matches: (+)** Right; **(-)** Wrong. Damages: **(1)** Damage to antenna; **(2)** Damage to legs; **(3)** Damage to antennae and legs; **(4)** Damage to wings; **(N)** No; **(Y)** Yes.

In the performance analysis, laboratories 1 and 5 presented the highest number of correct responses (83%, 10/12), whereas laboratory 3 showed the highest number of incorrect identifications (50%, 6/12). Participants in Laboratories 3, 4, and 6 were unable to differentiate between non-triatomines (i.e., predatory and phytophagous), demonstrating a basic lack of knowledge about the main morphological structure, the mouthparts (lips), for differentiating between phytophagous, predatory, and hematophagous insects.

The species with the greatest morphological similarity, such as *R. neglectus* and *R. brethesi*, as well as *T. maculata* and *T. pseudomaculata*, presented a higher frequency of errors. These errors reflect the taxonomic difficulties reported in the literature, especially for the genus *Rhodnius*, in which we found enormous morphological similarities between some species, making their identification difficult using dichotomous keys. Despite similar morphologies, these species may have distinct vectorial capabilities^11^ ^,^ ^12^.

The presence of structural damage in some specimens was not a determining factor for identification errors, as several species were correctly identified, as observed for *T. tibiamaculata* and *P. lignarius*.

Silva et al. also observed correct identification even in specimens with damaged morphological structures, highlighting that in routine identification laboratories, many insects arrive with some degree of morphological damage^12^. 

However, identification errors also occurred in undamaged individuals, indicating that similarity between closely related species is a more critical factor in the likelihood of errors than structural integrity. This finding suggests that striking diagnostic characteristics contribute to correct identification regardless of the state of conservation of the specimen.

The results of the current study reinforce the importance of ongoing training for participants responsible for triatomine identification, with an emphasis on species that are morphologically difficult to distinguish. Identification training using updated dichotomous keys, reference images, and high-resolution microscopy can reduce errors, particularly in species with similar morphological characteristics.

Difficulties related to the identification of species in the genus *Rhodnius* have been widely recognized since the first taxonomic studies in 1920^13^. Morphological similarity between species, especially external characteristics, continues to represent a significant challenge for entomological surveillance, which depends on accurate diagnoses. This scenario has become even more complex in recent decades, with the description of new species, such as *R. micki* Zhao, Galvão, & Cai, 2021, *R. montenegrensis* Rosa et al., 2012; *R*. *barretti* Abad-Franch et al., 2013; *R. marabaensis* Souza et al., 2016; and *R. taquarussuensis* Rosa et al., 2017, expanding the known diversity of the genus and making it more difficult to distinguish between closely related taxa.

The greatest difficulties occurred between *R. neglectus*, *R. brethesi*, *Rhodnius prolixus* Stål, 1859, and *R. nasutus*, which are often confused with each other. Switches were also recorded between *T. pseudomaculata* and *T. maculata*, as well as between *T. brasiliensis* and *T. melanica* Costa, Argolo, & Felix, 2006, which are species with morphological similarities.

The following ten triatomine species were confused with the panel species: *T. melanica*, *Triatoma delpontei* Romana & Abalos, 1947, *T. melanocephala*, *Triatoma matogrossensis* Leite & Borba,1953, *T. petrocchiae*, *R. prolixus*, *R. nasutus*, *Rhodnius robustus* Larrousse, 1927, *Rhodnius milesi* Carcavallo et al., 2001, and *Panstrongylus martinezorum* Ayala, 2009. Notably, the species *R. prolixus* and *P. martinezorum* are currently not recorded in Brazil. *Rhodnius prolixus* is distributed throughout northern South America and Central America, and is the main vector in Venezuela, as is *P. martinezorum*, which is currently restricted to that country. Regarding the identification of *R. milesi*, this species was formally synonymized with *R. neglectus* in 2024^14^.

The errors observed in the identification of triatomines during EQA can be attributed mainly due to the morphological similarity between closely related species, such as *T. maculata* and *T. pseudomaculata*, which were confused four times, and the species of the genus *Rhodnius* (*R. neglectus* and *R. brethesi*), which were confused five times. Training limitations, such as one-off and non-continuous training, which cause knowledge to be partially retained and lost over time; the lack of taxonomic updates with more recent keys containing descriptions of new species; and the lack of reference material for direct comparisons, are the factors that can most impact the performance of laboratories when identifying triatomines. 

Species identification by participants varied according to the clarity of the observed morphological characteristics. In species with more evident diagnostic structures, the rate of correct identification was higher^1^. Thus, striking diagnostic characteristics tend to favor correct identification because they reduce ambiguities during the recognition process^12^. 

These factors reinforce the need for ongoing training and provision of complementary tools to increase diagnostic accuracy within the scope of entomological surveillance.

Isolated cases of missing responses for species or sex were observed (4%, 3/72), as well as changes in the identification of predatory and phytophagous insects (5.5%, 4/72).

Were emphasize that EQA is not a definitive process; it reflects the performance of professionals only when performed. Therefore, EQA should be understood as an ongoing process capable of monitoring changes in laboratories, incorporating updates on geographic distribution, and identifying new triatomine species^15^.

EQA allowed us to conclude that although the identification of triatomines is satisfactory for most species, significant difficulties persist in distinguishing morphologically similar taxa. 

These findings reinforce the need for periodic training, use of updated taxonomic tools, and integration of complementary methods to improve diagnostic accuracy. Such measures are essential to strengthen entomological surveillance and timely responses to the presence of *T. cruzi* vectors in endemic areas^1^.

In summary, the results highlight the the need to strengthen and expand the training and standardization of entomological identification at a national scale, consolidating the strategic role of the reference laboratory as a central axis for the coordination and qualification of health surveillance actions.

## Data Availability

Research data is only available upon request.
